# Impact of Laparoscopic Sleeve Gastrectomy on Menstrual Regularity and Spontaneous Pregnancy in Morbidly Obese Women: A Retrospective Cohort Study

**DOI:** 10.3390/medicina62010191

**Published:** 2026-01-16

**Authors:** Zekai Serhan Derici, Tufan Egeli, Cihan Agalar, Suleyman Özkan Aksoy, Koray Atila

**Affiliations:** Department of Surgery, Faculty of Medicine, Dokuz Eylul University, 35340 Balcova, Turkey; tufanegeli@gmail.com (T.E.); cihanagalar@hotmail.com (C.A.); suleyman.aksoy@yahoo.com (S.Ö.A.); koray.atila@deu.edu.tr (K.A.)

**Keywords:** bariatric surgery, sleeve gastrectomy, obesity, infertility, pregnancy, menstrual cycle, contraception

## Abstract

*Background and Objectives*: Obesity is a major contributor to female reproductive dysfunction, frequently resulting in menstrual irregularity, anovulation, and subfertility. Bariatric surgery improves metabolic health; however, its effect on reproductive outcomes—particularly the shift from assisted to spontaneous conception—remains incompletely defined. This study aimed to evaluate the impact of laparoscopic sleeve gastrectomy (LSG) on menstrual cycle regularity and spontaneous pregnancy rates in women of reproductive age. *Materials and Methods*: This retrospective observational study included 52 women aged 18–40 years who underwent LSG between January 2013 and October 2017. Self-reported menstrual history, as documented during routine preoperative assessment in the electronic medical records, and reproductive outcomes (including spontaneous and assisted conception) were compared between the preoperative and postoperative periods. The median follow-up duration was 38 months. *Results*: A significant improvement in menstrual regularity was observed (46.2% to 94.2%, *p* < 0.001). Among women attempting conception, 10/15 (66.7%) achieved spontaneous pregnancy; one conceived via ART. Notably, 57.1% of all pregnancies occurred within the first 12 months post-surgery, including three unintended conceptions. Additionally, among women who conceived spontaneously, four had a history of requiring assisted reproductive technologies (ART), including two who had previously failed to conceive despite ART treatment. *Conclusions*: LSG is associated with significant normalization of menstrual cycles and a qualitative shift toward spontaneous conception in morbidly obese women. The rapid return of fertility, which may exceed patient awareness, underscores the importance of comprehensive perioperative counseling regarding effective contraception to prevent unintended pregnancies during the active weight-loss phase.

## 1. Introduction

The global prevalence of obesity is escalating, with a particularly steep rise observed among women of reproductive potential [1]. Beyond its well-established cardiovascular and metabolic sequelae, obesity significantly impairs female reproductive physiology [2]. Pathologies such as menstrual irregularity, anovulation, subfertility, and compromised pregnancy outcomes are highly prevalent in this demographic. These dysfunctions are frequently driven by a complex interplay of insulin resistance, chronic low-grade inflammation, and dysregulation of the hypothalamic–pituitary–ovarian axis [3].

Currently, bariatric surgery remains the most efficacious intervention for morbid obesity, offering sustained weight reduction and the amelioration of comorbidities [4]. Crucially, these benefits extend to the reproductive system. Post-surgical weight loss is strongly correlated with the resumption of regular menstruation and the recovery of ovulation, outcomes largely linked to enhanced insulin sensitivity and the rebalancing of sex steroid hormones [5,6]. Recent systematic reviews substantiate these findings, confirming a positive correlation between surgically induced weight loss and the reversal of obesity-related infertility [7,8].

However, the rapid improvement in fertility potential may not be immediately recognized by patients. Many women continue to rely on their preoperative history of infertility, erroneously assuming that conception remains unlikely. This misconception often contributes to the neglect of contraception and results in unintended pregnancies during the early postoperative period [9].

Conception during the active catabolic phase following surgery poses significant risks. The first 12 to 18 months post-operation are characterized by rapid weight loss and potential micronutrient deficits, which can compromise both maternal health and fetal development [10]. Therefore, current clinical guidelines and consensus reports strongly advocate for delaying pregnancy during this window to ensure metabolic and nutritional stability [7,11].

The present study aimed to evaluate the effects of laparoscopic sleeve gastrectomy (LSG) on menstrual cycle regularity and spontaneous pregnancy rates in women of reproductive age with morbid obesity. By comparing reproductive outcomes before and after surgery, this study seeks to evaluate the impact of bariatric surgery on fertility potential and to highlight the clinical necessity of early reproductive counseling and effective contraception in the perioperative period.

## 2. Materials and Methods

### 2.1. Study Design and Patient Selection

This retrospective observational study was conducted at the Department of General Surgery, Dokuz Eylul University Faculty of Medicine. We reviewed the clinical records of patients who underwent laparoscopic sleeve gastrectomy (LSG) for morbid obesity between January 2013 and October 2017. During the study period, LSG was the only bariatric procedure performed at our institution; therefore, heterogeneity attributable to procedure type was inherently limited, which helped minimize confounding related to procedure-related variability.

The initial screening identified 246 patients who underwent LSG during the study period. Of these, 178 were female. To evaluate the specific impact of LSG on fertility, we included women of reproductive age, defined as 18 to 40 years. Patients outside this age range (*n* = 99) were excluded. Additional exclusion criteria included incomplete clinical data or missing follow-up documentation (*n* = 27). After applying these criteria, the final cohort consisted of women aged 18–40 years who had undergone LSG and had complete follow-up data (*n* = 52).

### 2.2. Surgical Procedure

All surgical interventions were performed using a standardized laparoscopic sleeve gastrectomy technique, adhering to the institutional protocols. The procedure involved the longitudinal resection of the stomach starting from the antrum to the angle of His using endoscopic staplers. Following resection, the staple line was reinforced with a continuous seromuscular suture using an absorbable barbed suture (V-Loc™). No concurrent gynecological or other abdominal surgical procedures were performed during the index operation.

### 2.3. Data Collection

Baseline demographic and clinical data (age, body mass index (BMI), comorbidities, and preoperative reproductive history) were retrospectively extracted from the hospital’s electronic medical record system. Menstrual history, contraceptive use, and fertility-related information had been routinely obtained and documented as part of the standardized preoperative bariatric surgery evaluation; these contemporaneous preoperative records were used to classify preoperative menstrual regularity (see Section 2.4). Postoperative reproductive outcomes (pregnancy intention, conception method, and pregnancy occurrence) were retrieved from routine postoperative follow-up documentation available in the electronic medical record. Data were checked for completeness and consistency, and any discrepancies were resolved by re-review of the source records.

### 2.4. Outcome Measures and Definitions

The primary outcomes were changes in menstrual regularity and pregnancy.

Menstrual Regularity: Menstrual status was evaluated based on detailed menstrual history recorded in the electronic medical record as part of the preoperative bariatric surgery evaluation. In accordance with standard clinical definitions (FIGO criteria), a “regular menstrual cycle” was defined as menstruation occurring at intervals of 24–38 days, with cycle-to-cycle variation not exceeding 7–9 days [12]. Menstrual patterns falling outside these parameters (frequent, infrequent, or variable) were classified as irregular.

Pregnancy Outcomes: Reproductive outcomes were categorized by the mode of conception. Spontaneous pregnancy was defined as conception achieved without the use of any assisted reproductive technologies (ART) or ovulation induction, confirmed by a positive serum β-hCG test. Pregnancies resulting from in vitro fertilization (IVF) or intrauterine insemination (IUI) were classified as ART conceptions.

### 2.5. Statistical Analysis

Data analysis was performed using IBM SPSS Statistics (Version 30.0). Continuous variables were presented as mean ± standard deviation (SD) or median (min–max). Categorical variables were expressed as frequencies (*n*) and percentages (%). Preoperative and postoperative menstrual patterns were compared using the McNemar test (for paired nominal data). In cases where cell counts were insufficient, the exact binomial version of the McNemar test was applied. A *p*-value < 0.05 was considered statistically significant.

### 2.6. Ethical Approval

The study protocol was approved by the Non-Interventional Clinical Research Ethics Committee of Dokuz Eylul University (Approval No: 2025/15-09 Date: 7 May 2025).

## 3. Results

### 3.1. Baseline Characteristics

The final analysis included 52 women of reproductive age. The median follow-up duration was 38 months (range: 6–62 months). The mean age of the cohort was 30.2 ± 6.3 years. Preoperatively, the mean body weight was 119.0 ± 15.6 kg, and the median BMI was 42.9 kg/m^2^ (range: 36–62). The prevalence of obesity-related comorbidities was relatively low in this young cohort; hypothyroidism was present in 11.5% (*n* = 6), diabetes mellitus in 5.8% (*n* = 3), hypertension in 3.8% (*n* = 2), and pulmonary disease in 3.8% (*n* = 2) of the patients. Baseline demographic and clinical characteristics are summarized in Table 1.

### 3.2. Menstrual Cycle Regularity

A significant improvement in menstrual pattern was observed following LSG. Before surgery, 53.8% of patients (*n* = 28) reported irregular menstrual cycles. Postoperatively, menstrual regularity was resumed in 26 of these 28 patients (92.9%). Conversely, only one patient with a previously regular cycle developed irregularity after surgery. Overall, 94.2% of the cohort (*n* = 49) reported regular cycles at follow-up. This change represented a statistically significant normalization of menstrual function (McNemar exact test, *p* < 0.001).

### 3.3. Reproductive Outcomes

#### 3.3.1. Preoperative Fertility History

Prior to surgery, 27 women reported a history of attempting conception. Of these, 10 achieved spontaneous pregnancy. Assisted reproductive technologies (ART) were utilized by 10 patients; this resulted in pregnancy in 5 cases, while 5 cases were unsuccessful. Seven patients were unable to conceive despite having a pregnancy intention during the preoperative period.

#### 3.3.2. Contraceptive Utilization

Analysis of contraceptive practices revealed that preoperatively, 12 women (23.1%) were using contraception. The methods included intrauterine devices (IUD) (*n* = 4), oral contraceptives (*n* = 2), barrier methods (*n* = 2), tubal ligation (*n* = 2), and withdrawal/calendar methods (*n* = 2). Postoperatively, the number of women using contraception increased to 17 (32.7%). The distribution of postoperative methods was as follows: barrier methods (*n* = 5), IUDs (*n* = 4), oral contraceptives (*n* = 3), tubal ligation (*n* = 3), and withdrawal/calendar methods (*n* = 2). The majority of the cohort (67.3%) reported no active contraceptive method during the postoperative follow-up.

#### 3.3.3. Postoperative Pregnancy Outcomes

During the postoperative follow-up period, 15 patients expressed an intention to conceive. Of this cohort, 10 achieved spontaneous pregnancy (66.7%). One patient—notably with a history of failed preoperative ART attempts—successfully conceived via ART. The remaining four patients attempted natural conception but were unable to conceive spontaneously during the study period (26.7%). Additionally, spontaneous pregnancy occurred in three patients who reported no active intention to conceive. In total, 14 patients became pregnant during the study.

A sub-analysis of the women who attempted and achieved spontaneous pregnancy postoperatively revealed that 4 of them had a documented history of requiring Assisted Reproductive Technologies (ART) prior to surgery. Specifically, 2 of these patients had previously conceived only via IVF, while the other 2 had a history of failed IVF attempts. The fact that all 4 achieved spontaneous pregnancy postoperatively highlights a potential improvement in fertility, independent of preoperative fertility status.

#### 3.3.4. Unintended Pregnancies

Three spontaneous pregnancies occurred in patients who reported no intention to conceive during the postoperative follow-up.

**Figure 1:**
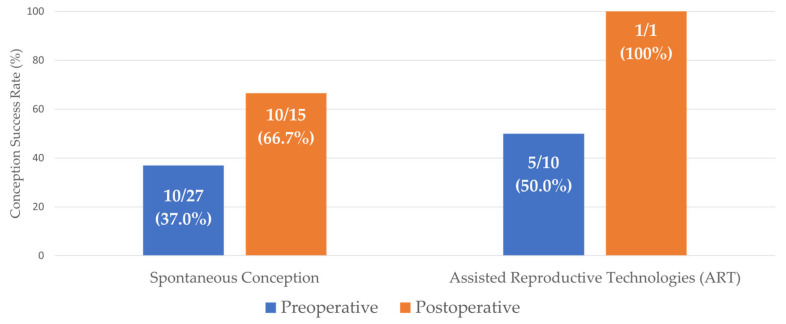
Comparison of conception success rates before and after bariatric surgery among women with pregnancy intention. The graph displays the success rates of spontaneous conception and assisted reproductive technologies (ART). Bars represent the preoperative period and postoperative period, respectively. Data labels indicate the number of successful pregnancies relative to the total number of patients attempting to conceive (*n*/*N*, %). Note: Three spontaneous pregnancies occurring in women without postoperative pregnancy intention were excluded from this analysis to ensure comparability.

The comparison of reproductive outcomes and conception methods between the preoperative and postoperative periods is illustrated in Figure 1. The success rate of spontaneous conception increased from 37.0% preoperatively to 66.7% postoperatively. Concurrently, the reliance on ART diminished; while 10 patients required ART preoperatively (with a 50% success rate), only one patient required ART postoperatively, achieving successful conception (100%) (Figure 1).

### 3.4. Timing and Clinical Status of Postoperative Conception

Analysis of the surgery-to-conception interval indicated a rapid return of fertility. The median time from surgery to conception was 12 months (range: 3–48 months). Notably, 8 out of 14 pregnancies (57.1%) occurred within the first 12 months post-surgery, a period generally characterized by active weight loss. Among the three unintended pregnancies, conception occurred as early as 5 months postoperatively (with the others occurring at 12 months), suggesting an early improvement in ovulatory function even before weight stabilization is achieved.

Regarding the metabolic status at the time of conception, the clinical profile of these 14 women (11 with intention, 3 unintended) had improved. The mean BMI at the onset of pregnancy was 29.4 ± 4.0 kg/m^2^ (median: 28.8 kg/m^2^), and the mean body weight was 80.4 ± 13.9 kg. This corresponded to a mean excess weight loss (EWL) of 80.4% ± 18.2% (median: 81.3%; range: 55.4–117.8%).

## 4. Discussion

The present study indicates that laparoscopic sleeve gastrectomy (LSG) is associated with substantial improvements in the reproductive health of women of reproductive age. Our findings suggest improvements in reproductive parameters, including menstrual regularity and a shift toward spontaneous conception. Crucially, the occurrence of pregnancies in women with no stated intention to conceive highlights a clinically important and potentially under-recognized consequence of bariatric surgery: the rapid return of fecundity often outpaces patient awareness.

Menstrual irregularity is a hallmark of obesity-related reproductive dysfunction, frequently driven by the interplay of insulin resistance, hyperinsulinemia, and the dysregulation of the hypothalamic–pituitary–ovarian (HPO) axis [13]. Surgical weight loss has been consistently shown to ameliorate these metabolic derangements, thereby facilitating the resumption of spontaneous ovulation [5,14]. In our cohort, the marked increase in regular menstrual cycles (from 46.2% to 94.2%) supports this physiological framework. This finding is consistent with prior literature suggesting that metabolic improvements after bariatric surgery may contribute to improved ovulatory function in obesity-related anovulation [15].

Beyond menstrual cycle regularization, the nature of conception exhibited a marked shift post-surgery. Preoperatively, patients attempting pregnancy frequently faced subfertility and often required assisted reproductive technologies (ART), with limited success. In our cohort, among the patients who attempted natural conception postoperatively, 66.7% (10/15) successfully achieved spontaneous pregnancy. Conversely, in the postoperative period, spontaneous conception became the predominant mode of pregnancy, largely reducing the reliance on ART. Although the varying denominators preclude a direct statistical comparison between the two periods, this descriptive reversal suggests a clinically relevant improvement in documented increased conception rates and live birth rates following metabolic surgery, particularly in women with a history of infertility [16,17,18,19]. Notably, we observed instances of spontaneous conception even in patients with a documented history of unsuccessful IVF attempts, highlighting a clinically relevant outcome in this specific subgroup.

A critical observation in our study is that pregnancy was not exclusive to women actively seeking conception. Three spontaneous pregnancies occurred in patients who explicitly reported no pregnancy intention during the postoperative follow-up. These cases underscore a pervasive clinical pitfall: women with a long history of obesity-related infertility may harbor the erroneous assumption that their inability to conceive persists post-surgery. Consequently, they may neglect contraception [20]. This suggests that reproductive potential may improve rapidly—often before significant weight loss is fully achieved—highlighting the unreliability of past infertility as a predictor of future sterility [21,22].

In addition to physiological recovery, the psychosocial and behavioral impact of surgery plays a pivotal role in reproductive outcomes. Weight loss following bariatric surgery is frequently associated with improved body image, self-confidence, and sexual function [23]. Enhanced libido and sexual satisfaction, when combined with the resumption of ovulatory function, may create a conducive environment for unintended pregnancy if effective contraception is not employed. From this perspective, the unintended pregnancies observed in our cohort may reflect the synergistic effect of biological fertility recovery and increased sexual activity, rather than metabolic recovery alone.

However, the timing of conception following bariatric surgery remains a subject of clinical debate. Current consensus guidelines generally advise delaying pregnancy for 12 to 18 months post-surgery to avoid the active catabolic phase, which poses theoretical risks regarding maternal nutritional status and fetal growth [24,25,26,27]. Our findings reveal a significant divergence from these recommendations in real-world practice; notably, 57.1% of pregnancies in our cohort occurred within this critical first postoperative year, with one unintended conception occurring as early as the fifth month. This suggests that fertility often rebounds faster than anticipated, potentially exposing the fetus to a period of dynamic metabolic flux where optimal nutritional repletion is challenging [28]. While some data specific to sleeve gastrectomy indicate that long-term obstetric outcomes may not be severely compromised even in early pregnancies, avoiding conception during the rapid weight loss phase remains the prudent clinical recommendation [7,29].

These findings underscore the importance of a proactive approach to contraceptive counseling. Literature suggests that despite the known return of fertility, postoperative contraceptive adherence remains inconsistent [16,30,31]. Given the potential for malabsorption affecting oral contraceptives (though less of a concern in sleeve gastrectomy compared to bypass procedures) and the need to minimize user-dependent failure, long-acting reversible contraceptives (LARC), such as intrauterine devices or implants, are increasingly preferred [24,30]. The urgency of this approach is highlighted by our observation that postoperative contraceptive utilization remained low (32.7%), with a persistent reliance on user-dependent methods rather than high-efficacy options. This finding suggests that standard counseling may not be fully effective in bridging the gap between clinical recommendations and patient behavior. Our data reinforce the necessity of establishing a robust contraceptive plan—ideally before the surgery takes place—to bridge the gap between the return of fertility and the safe timing for pregnancy.

Our study has several limitations inherent to its design. The retrospective nature and single-center setting limit the generalizability of the findings. Additionally, the exclusion of eligible patients due to missing data or incomplete follow-up documentation may introduce potential selection bias. Menstrual regularity was determined via patient self-report rather than objective hormonal assays or ovulation tracking. Furthermore, the relatively small sample size precludes extensive subgroup analyses. However, the consistency of the data—showing a clear trend toward menstrual normalization and spontaneous conception—provides a coherent clinical narrative that supports current recommendations.

Despite the growing body of literature confirming fertility restoration, recent evidence suggests that patients frequently remain inadequately informed about these rapid reproductive changes. Studies indicate that formal documentation of contraceptive counseling is often absent in clinical records, and a significant proportion of surgeons do not routinely discuss reproductive planning with patients [20,32,33]. A recent scoping review further highlights systemic deficiencies in provider knowledge and comfort regarding this counseling [34]. Consequently, many women continue to neglect contraception during the rapid weight-loss phase, often due to persistent misconceptions regarding their fertility status [9]. This persistent gap between scientific knowledge and clinical counseling underscores the urgency of raising awareness among clinicians to prevent unintended pregnancies and protect fetal health.

Future studies should build on these findings using prospective, multicenter designs with standardized and objective assessments of reproductive function. In addition to clinical outcomes, incorporating ovulatory markers and hormonal profiling (e.g., mid-luteal progesterone and key gonadotropin/androgen parameters), supported when feasible by structured ultrasound-based follicular assessment, would reduce reliance on self-reported menstrual patterns. Future protocols should also capture body composition and nutritional/metabolic parameters during the rapid weight-loss phase, as BMI alone may be insufficient. Comparative studies across bariatric procedures with different mechanistic profiles (restrictive vs. malabsorptive/combined) may clarify whether fertility-related outcomes and contraceptive considerations, including potential effects on oral medication bioavailability, differ by procedure type.

## 5. Conclusions

Laparoscopic sleeve gastrectomy in morbidly obese women was associated with improved menstrual regularity and an increased rate of spontaneous conception in our cohort. Fertility may recover earlier than anticipated, leading to pregnancies even in the absence of intention. These findings highlight the critical need for a continuum of reproductive care. Comprehensive counseling involving bariatric surgeons, obstetrician-gynecologists, and dietitians should be initiated preoperatively to address pregnancy timing and effective contraception. Such a multidisciplinary approach is essential to ensure that the improvement in fertility results in planned, healthy pregnancies rather than unintended ones during the high-risk postoperative period.

## Figures and Tables

**Table 1:** Baseline demographic and clinical characteristics of the study population.

Characteristic	Value (*n* = 52)
Age (years), mean ± SD	30.2 ± 6.3
Follow-up duration (months), median (range)	38 (6–62)
Height (cm), mean ± SD	163.7 ± 6.3
Preoperative weight (kg), mean ± SD	119.0 ± 15.6
Preoperative BMI (kg/m^2^), median (range)	42.9 (36–62)
Comorbidities	
Diabetes mellitus, *n* (%)	3 (5.8%)
Hypertension, *n* (%)	2 (3.8%)
Pulmonary disease, *n* (%)	2 (3.8%)
Hypothyroidism, *n* (%)	6 (11.5%)
Preoperative Menstrual Pattern	
Regular cycle, *n* (%)	24 (46.2%)
Irregular cycle, *n* (%)	28 (53.8%)

BMI: body mass index; SD: standard deviation.

## Data Availability

The data sets used and/or analyzed during the current study are available from the corresponding author upon reasonable request.

## Abbreviations

The following abbreviations are used in this manuscript:

ART	Assisted Reproductive Technologies
BMI	Body Mass Index
EWL	Excess Weight Loss
FIGO	International Federation of Gynecology and Obstetrics
HPO	Hypothalamic–Pituitary–Ovarian
IUD	Intrauterine Devices
IUI	Intrauterine Insemination
IVF	In Vitro Fertilization
LARC	Long-Acting Reversible Contraceptives
LSG	Laparoscopic Sleeve Gastrectomy
SD	Standard Deviation
β-hCG	Beta-human Chorionic Gonadotropin
